# 2024 reviewer index

**DOI:** 10.26508/lsa.202503209

**Published:** 2025-01-17

**Authors:** Eric Sawey

**Affiliations:** Cold Spring Harbor Laboratory , Cold Spring Harbor, NY, USA

## Abstract

The LSA Editors thank these scientists for sharing their time and expertise while reviewing manuscripts in 2024. We sincerely appreciate their support.

Agallou, Maria

Aguilera Castrejon, Alejandro

Akay, Alper

Akira, Shizuo

Alam, Amer

Alexander, Matthew

Algood, Holly M

Allain, Frederic H

Allen, Scott P

Allred, David R

Almouzni, Genevieve G

Alonso, Sylvie

Amatruda, James

Amini, Hajar

Andrisani, Ourania M

Antoniou, Anna

Arndt, Karen M

Aspenström, Pontus

Atfi, Azeddine

Atwood, Scott

Augenlicht, Leonard

Azami, Takuya

Bénard, Giovanni

Bachovchin, Daniel A

Baehr, Wolfgang

Baek, Lily

Balayssac, David

Baldwin, Enoch

Bajaj, Teena

Ballif, Bryan A

Baoli, Yao

Baralle, Francisco

Barau, Joan

Bardoni, Barbara

Barisic, Marin

Baritaki, Stavroula

Barral, Duarte

Barrientos, Antoni

Baserga, Susan J

Bastepe, Murat

Bates, Gillian

Bayly, Philip

Beckel, Jonathan

Beis, Konstantinos

Bellusci, Saverio

Beltra, Marc

Benetton, Maddalena

Berezin, Mikhail

Bernecky, Carrie

Bernstein, Steven

Berntsson, Ronnie P

Bertolin, Giulia

Bhargava, Nishtha

Bhattacharyya, Suvendra N

Bhuiyan, Zahurul

Biggane, Joseph

Birchenough, George

Blüthgen, Nils

Blades, Farrah

Blanton, Robert

Bleichert, Franziska

Blin, Guillaume

Boag, Peter R

Boccaccio, Graciela

Boeda, Batiste

Bogenhagen, Daniel F

Borggaard, Xenia Goldberg

Bosque-Pardos, Alberto

Bou-Gharios, George

Boucher, Dave

Bourc’his, Deborah

Bröker, Barbara M

Bradbury, Allison M

Brasch, Julia

Braun, Thomas

Brazauskas, Pijus

Brejová, Broňa

Brenner, Charles

Brodin, Birger

Brody, Jonathan

Brown, Amanda

Buchanan, Susan K

Buchberger, Alexander

Budimirovic, Dejan

Buettner, Sabrina

Bullock, Simon L

Bulyk, Martha L

Busserolles, Jérome

Buxbaum, Joel

Calado, Dinis P

Calderon de Anda, Froylan

Cambillau, Christian

Campos, Samuel K

Candi, Eleonora

Cantu, Claudio

Carlton, Jeremy G

Carty, Ben

Castrillo, Antonio

Chacinska, Agnieszka

Chandwani, Rohit

Charrier, Cecile

Chen, Bin

Chen, Ching-Yi

Chen, Jun-An

Chen, Sheng-Hong

Chen, Yi-Guang

Cheng, Jung Hwa (Susan)

Cheng, Shaoping

Cheng, Zhukuan

Chernyavsky, Igor

Chlopicki, Stefan

Christie, Graham

Christie, Peter J

Cingolani, Gino

Ciosk, Rafal

Cipollina, Chiara

Cirulli, Vincenzo

Clarke, Duncan J

Clyne, Alisa M

Coates, Philip J

Cocquet, Julie

Cocucci, Emanuele

Collins, Sean

Colpitts, Che C

Colque, Antonella

Comoletti, Davide

Conigliaro, Alice

Cook, Jeanette G

Cook, Peter R

Copp, Andrew

Coulson, Elizabeth J

Coux, Olivier

Coyne, Alyssa Nicole

Cristoffels, Vincent M

Cubero, Ryan

Cui, Jianmin

Cumano, Ana

Czogalla, Aleksander

D’Amours, Damien

Daly, Ann

Das, Sonali

Dash, Rupesh

Davidson, Gary

Davies, Matthew

De Camilli, Pietro V

De Meester, Ingrid

De Stefani, Diego

De Swart, Rik L

De Wit, Elzo

Deans, Michael

Decker, Thomas

Decroly, Etienne

Delev, Daniel

Deme, Justin

Deniz, Özgen

Dernburg, Abby

Descoteaux, Albert

Despotovic, Jovana

Dettmer, Ulf

Di Cesare Mannelli, Lorenzo

Didonna, Alessandro

DiFiglia, Marian

Dimmer, Kai S

Dittmer, Dirk P

Diz-Muñoz, Alba

Docampo, Roberto

Doitsidou, Maria

Dong, Zheng

Donlin-Asp, Paul G

Dooley, Steven

Dorhoi, Anca

Dormann, Dorothee

Dou, Yongchao

Douzi, Baddreddine

Downs, Karen

Downward, Julian

Dreveny, Ingrid

Drew, David

Drubin, David

DuBois, Raymond N

Dumont, Nicolas A

Durand, Bénédicte

Dutcher, Susan K

Dynlacht, Brian D

Ellis, Stephanie

Emiliani, Stéphane

Erickson, Robert P

Eriksson, Emily M

Erlacher, Matthias

Espona-Fiedler, Margarita

Essers, Marieke A

Faissner, Andreas

Fandrey, Joachim

Farahbod, Marjan

Farber, Steven

Fardous, Ali

Faria-Pereira, Andreia

Farmer, Mark A

Faundez, Victor

Febbraio, Maria

Ferguson, Neil M

Fernández-Checa, José Carlos

Ferrer, Jorge

Ferri, Alberto

Field, Mark C

Field, Michael

Fierz, Beat

Fingleton, Barbara

Fisher, Robert P

Fitzgerald, Katherine A

Flynn, Jullien

Fodde, Riccardo

Forst, Christian V

Foulquier, François

Francastel, Claire

Francia, Maria

Francia, Sofia

Fratti, Rutilio A

Frazer, Kelly A

Freeman, Willard

Friedman, Jonathan

Fry, Andrew M

Fujita, Masatoshi

Fukada, So-Ichiro

Funato, Kouichi

Götz, Magdalena

Galjart, Niels

Galli, Francesco

Gallo, Luciana

Ganesh, Bhanu P

Garapati, Kishore

Gardella, Thomas

Gascón, Sergio

Gehring, Niels H

Genetta, Thomas L

Genta, Ito

Gerber, André P

Ghosh, Avishek

Ghoshre, Arpita

Giacomello, Marta

Gilbert, Wendy V

Giliberto, Florencia

Gillet, Reynald

Ginhoux, Florent

Glaab, Enrico

Glass, David

Gloerich, Martijn

Gold, Michael R

Golde, Todd E

Gomis, Roger R

Gonatopoulos-Pournatzis, Thomas

Gorfe, Alemaheyu A

Gorrell, Mark D

Gorsope, Myriam

Goubert, Clément

Goud, Bruno

Gowrishankar, Swetha

Graier, Wolfgang F

Grant, Chris M

Grant, Heather

Groves, Matthew

Guarguaglini, Giulia

Gubbels, Marc-Jan

Guillen-Samander, Andres

Guo, Shuaiqi

Gupta, Mohona

Gussoni, Emanuela

Gutschner, Tony

Härtlova, Anetta

Hainer, Sarah

Hains, David S

Halberg, Nils

Han, Zeguang

Hanna, Jacob H

Hanna, Stephanie

Hansen, Scott B

Harland, Luke

Hartkoorn, Ruben C

He, Sicong

Hegde, Ramanujan

Henry, Michael D

Herlyn, Meenhard

Hernandez, Greco

Hescheler, Juergen

Hesselson, Daniel

Hetmanski, Joseph

Higashikuni, Yasutomi

Hilfiker, Sabine

Hinck, Lindsay

Ho, Diana M

Hoffmann, Werner

Hollywood, Mark

Holoch, Daniel

Horie, Minoru

Hristov, Borislav

Hu, Guang

Hu, Junjie

Huang, Hongda

Hughes, Jing W

Hunstad, David

Hyacinth, Hyacinth I

Igreja, Catia

Ikeda, Fumiyo

Ikui, Amy E

Ilkayeva, Olga

Imai, Yuzuru

Inaba, Kazuo

Ingolia, Nicholas

Irvine, Katharine

Itoh, Yoshifumi

Ivaska, Johanna

Iyer, Jyoti

Jékely, Gáspár

Jackson, Christopher B

Jain, Bhawik K

Janssen, Bert

Janssens, Sophie

Jaumouillé, Valentin

Jenq, Robert R

Jeyaseelan, Samithamby

Jhunjhunwala, Suchit

Jiang, Xue-Yan

Jin, Tian

Jo, Young Ah

Johansson, Malin E

Johnson, Lance

Jones, Matt

Jordheim, Lars P

Jourdain, Alexis A

Kabir, Ashraf Ul

Kallas, Dania

Kallijärvi, Jukka

Kanemaki, Masato T

Karner, Courtney M

Kass, David A

Katanaev, Vladimir L

Kato, Hiroki

Keenan, Christine

Kellaway, Sophie G

Kelly, Robert G

Kerr, Ian D

Kim, Boa

Kim, Boe-Hyun

Kim, Jun Yong

Kim, Yoosik

Kitajima, Tomoya S

Kizil, Caghan

Kläsener, Kathrin A

Knaus, Petra

Knobloch, Marlen

Knop, Michael

Kolch, Walter

Kolias, Georges

Kollias, George

Konno, Tasuku

Kooistra, Susanne M

Kopp, Janel L

Korbel, Jan O

Korolchuk, Viktor I

Koseki, Haruhiko

Kouskoff, Valerie

Krashes, Michael

Kretschmar, Kai

Krishnamoorthy, Raghu R

Kumar, Vinod

Kumari, Sudha

Kuna, Marija

Kuo, Yi-Tzu (Claudia)

Laabel, Maisem

Laidlaw, Brian J

LaMarca, Babette D

Landfear, Scott M

Landreth, Gary

Lappalainen, Pekka

Lausen, Jörn

Lavelle, Ed C

Lavia, Patrizia

Leander, Brian

Lederkremer, Gerardo Z

Lee, Chien-Kuo

Lee, Chun G

Lee, Tom V

Lee, Youn-Bok

Lehtinen, Sonja

Leichert, Lars I

Lesch, Klaus-Peter

Levi, Benjamin

Li, Chia-Wei

Li, Henry James (Xiaotao)

Li, Huilin

Li, Liwu

Liakath-Ali, Kif

Lian, Qizhou

Liccardi, Gianmaria

Lichten, Michael J

Lichterfeld, Mathias

Liesa, Marc

Lieske, John

Lightowlers, Robert N

Lin, Chih-Chung

Lin, Wensheng

Liu, Ao

Liu, Chunyu

Liu, Shujun

Lo, Su H

Lohi, Olli

Lopez, Pablo Hector Horacio

Lowy, Douglas R

Lubberts, Erik

Lucassen, Paul J

Luebke, Joachim

Lusser, Alexandra

Müller-Dahlhaus, Florian

Ma, Hoi Tang

Ma, Yingze

Maciel, Thiago T

Macurek, Libor

Magalhaes, Eduardo

Maiato, Helder

Mansky, Kim

Mantamadiotis, Theo

Marchant, David J

Margueron, Raphael

Markowitz, Sanford

Marks, Michael S

Martin, Lee

Mastrianni, James

Matsushita, Kazufumi

Mattedi, Francesca

Matteoli, Gianluca

Mattick, John S

Maxfield, Frederick R

McClane, Bruce A

McCulloch, Chris A

McMaster, Christopher R

McNamara, Niamh

Meagher, Robert

Mehta, Gunjan

Meijer, Dimphna

Meijer, Wilfried J

Mendez, Mariela

Micheva, Kristina D

Milan, Enrico

Mildner, Alexander

Millán, Jaime

Miller, Kyle M

Millet, Arnaud

Mishina, Yuji

Mitani, Shohei

Miyoshi, Takushi

Mladenov, Emil

Modahl, Cassandra M

Mollen, Kevin

Monahan, Kevin

Montag, Judith

Moos, Torben

Mor, Danielle E

Morgan, Ethan L

Mori, Kohji

Morrison, Ciaran

Morton, AJ

Mota, Maria M

Mudher, Amritpal

Mueller, Jacob

Mukhopadhyay, Somshuvra

Munro, Sean

Murphy, Robert F

Murphy, Robyn M

Murray, Peter

Nakajima, Osamu

Nakano, Hiroyasu

Narunsky, Aya

Natarajan, Siva Kumar

Nebert, Daniel W

Nellist, Mark

Nelson, Sacha B

Neogi, Ujjwal

Neuman, Benjamin W

Niarakis, Anna

Nicholls, Thomas

Niemi, Natalie M

Nilsson, Jakob

Noel, Richard

Noh, Kyung-Min

Nordlund, Jessica

Norris, Adam

Noto, Jennifer M

O’Toole, George

Obara, Christopher

Oliveras, Anna

Olkkonen, Vesa M

Ono, Shoichiro

Orlandi, Cesare

Otsuka, Shotaro

Owens, Nick

Pöling, Jochen

Palis, James

Panday, Arvind

Park, Sang Gyu

Parsons, Jason L

Patel, Smita

Patras, Kathryn

Pearson, Dustin D

Pease, James E

Pederson, Stephen M

Peled, Jonathan U

Pellman, David S

Peng, Xinxia

Pereira, Joao P

Perretti, Mauro

Persidsky, Yuri

Pertz, Olivier

Perucha, Esperanza

Pfrieger, Frank W

Piano Mortari, Eva

Piedra, Pedro

Piers, Tom

Pillai, Ramesh S

Pilz, Gregor

Pinar, Halit

Pinilla, Estéfano

Poisson, Johanne

Poterszman, Arnaud

Prakash, Hridayesh

Prasanth, Kannanganattu

Previtali, Stefano C

Prigent, Claude

Proikas-Cezanne, Tassula

Prudencio, Miguel

Puschel, Andreas W

Qian, Shu-Bing

Qin, Zhiqiang

Rabouille, Catherine

Raghavan, Malini

Raimundo, Nuno

Ramachandran, Rajesh

Ramachandran, Srinivas

Ramaswami, Mani

Ramezani-Rad, Parham

Randazzo, Paul A

Rao, Ponugoti V

Rao, Sadhna

Raote, Ishier

Ratcliffe, Colin D

Reboldi, Andrea

Redemann, Stefanie

Rees, Paul

Regnier-Vigouroux, Anne

Rehwinkel, Jan

Reichmann, Dana

Reiner, David J

Reiter, Lawrence T

Reyes Palomares, Armando

Richmond, Jillian M

Ridley, Anne J

Riezman, Howard

Robert, Francois

Rodrigues, Elsa

Rodrigues Muys, Bruna

Rohn, Jennifer L

Ron, David

Rooijakkers, Suzan S

Roos, Andreas

Rossi, Martina

Rothenberg, Ellen V

Roy, Debjyoti

Russo, Brian

Ruytinx, Pieter

Ruzzenente, Benedetta

Rybniker, Jan

Saba, Karim

Sablina, Anna A

Saeki, Keita

Saikia, Queen

Sahoo, Pabitra

Salinas, Patricia C

Saltiel, Alan

Sandri, Marco

Sanguinetti, Guido

Sanguinetti, Michael C

Saric, Amra

Sarnataro, Daniela

Sartori, Alessandro A

Sashittal, Palash

Sauer, David B

Sawitzki, Birgit

Sayer, John A

Schiebel, Elmar

Schinder, Alejandro

Schlegel, Amnon

Schlepckow, Kai

Scholey, Jonathan M

Schuberth, Hans-Joachim

Schwartz, Yulia

Schwarz, Roland

Scorrano, Luca

Scott, Patricia

Sedman, Juhan

Selvatici, Rita

Sen, Mehmet

Senavirathna, Indika

Sendler, Matthias

Sengupta, Sourya

Seo, Heewon

Seo, Young Ah

Sessa, Alessandro

Setlow, Peter

Shahbazi, Marta

Shaikh, Nader

Sharan, Shyam K

Sharma, Amit

Shen, Hongxing

Shi, De-Li

Shi, Yi

Shibata, Tatsuhiro

Shimada, Issei S

Shinohara, Akira

Shirihai, Orian S

Sigal, Michael

Sinnberg, Tobias

Slavoff, Sarah A

Smith, Adam

Snyder, Elizabeth

Solary, Eric

Soreq, Hermona

Spillane, Katelyn

Stahelin, Robert V

Stallmeyer, Birgit

Stanley, E Richard R

Stathopulos, Peter B

Stefan, Christopher J

Stemmann, Olaf

Stewart, Grant S

Stins, Monique

Stockmann, Christian

Storz, Gisela

Stow, Jennifer L

Straub, Monique

Strippoli, Raffaele

Strober, Warren

Strom, Amy R

Su, Xin-Zhuan

Sunnerhagen, Per

Supek, Fran

Suzuki, Tadaki

Suzuki, Yasuhiro

Suzuki, Yutaka

Swanson, Chad M

Szafranska, Karolina

Szostek-Mioduchowska, Anna

Szulzewsky, Frank

Tamarit, Jordi

Taneera, Jalal

Tartour, Eric

Taylor, Cormac T

Taylor, Gregory A

Taylor, Naomi

Taylor, Nicholas

Taymans, Jean-Marc

Techer, Hervé

Teschendorff, Andrew

Therrien, Martine

Tilley, Leann

Tollervey, David

Tong, Qingchun

Torrent, Marc

Trost, Matthias

Trowitzsch, Simon

Tsiairis, Charisios

Tsiokas, Leonidas

Turcan, Şevin

Ugalde, Cristina

Uliana, Federico

Ulitsky, Igor

Ungar, Daniel

Ungermann, Christian

Updike, Dustin

Van Damme, Philip

Van den Ameele, Jelle

Van Wijk, Sjoerd

Vance, Keith W

Vardi, Noga

Vargas, Pablo

Vashisht Gopal, YN

Verma, Akanksha

Verreault, Alain

Verzi, Michael P

Vicente-Manzanares, Miguel

Vidal, Miguel M

Villeneuve, Julien

Villunger, Andreas

Voss, Matthias

Wada, Ikuo

Wagner, Kay-Uwe

Wahling, Staffan

Walczak, Henning

Walker, Brian A

Walter, Jochen

Walter, Jorn

Wang, Hai

Wang, Hongbing

Wang, Xinxin

Wang, Yi

Wang, Yingxiang

Washburn, Michael

Wassmann, Katja

Weinkove, David

Wek, Ronald

Wen, Qi

West, John T

Weyand, Cornelia M

Wilk, Sherwin

Willert, Karl

Wilson, Danny W

Wilusz, Jeffrey

Wilusz, Jeremy E

Winner, Beate

Wirths, Oliver

Wiseman, RL

Witztum, Joseph L

Wolf, Dieter A

Wood, Charles

Wredenberg, Anna

Wulfing, Christoph

Xia, Huiming

Xiang, Guanjue

Xiao, Tianyao

Xiao, Xiangwei

Xu, Xuehua

Yalowich, Jack

Yamada, Soichiro

Yamagata, Takanori

Yamamoto, Shinya

Yan, Shunping

Yang, Fen

Yang, Juan

Yoon, Jaeho

Yoshimi, Akihide

Yu, Yong

Yu, Zhantao

Yuan, Guo-Cheng

Yun, Maximina

Zanetti, Maurizio

Zaugg, Judith B

Zegers, Mirjam

Zhang, Hong

Zhang, Jian

Zhang, Liye

Zhang, Yunxiao

Zhen, Yang

